# Inter-rater reliability of the QuIS as an assessment of the quality of staff-inpatient interactions

**DOI:** 10.1186/s12874-016-0266-4

**Published:** 2016-12-07

**Authors:** Ines Mesa-Eguiagaray, Dankmar Böhning, Chris McLean, Peter Griffiths, Jackie Bridges, Ruth M Pickering

**Affiliations:** 1 Medical Statistics Group, Faculty of Medicine, Southampton General Hospital, Mailpoint 805, Level B, South Academic Block, Southampton, SO16 6YD UK; 2 Southampton Statistical Sciences Research Institute & Mathematical Sciences, University of Southampton, Southampton, UK; 3 Faculty of Health Sciences, University of Southampton, Southampton, UK

**Keywords:** Weighted kappa, Random effects meta-analysis, QuIS, Collapsing, Averaging

## Abstract

**Background:**

Recent studies of the quality of in-hospital care have used the Quality of Interaction Schedule (QuIS) to rate interactions observed between staff and inpatients in a variety of ward conditions. The QuIS was developed and evaluated in nursing and residential care. We set out to develop methodology for summarising information from inter-rater reliability studies of the QuIS in the acute hospital setting.

**Methods:**

Staff-inpatient interactions were rated by trained staff observing care delivered during two-hour observation periods. Anticipating the possibility of the quality of care varying depending on ward conditions, we selected wards and times of day to reflect the variety of daytime care delivered to patients. We estimated inter-rater reliability using weighted kappa, κ_*w*_, combined over observation periods to produce an overall, summary estimate, ${\widehat{\upkappa}}_w$. Weighting schemes putting different emphasis on the severity of misclassification between QuIS categories were compared, as were different methods of combining observation period specific estimates.

**Results:**

Estimated ${\widehat{\upkappa}}_w$ did not vary greatly depending on the weighting scheme employed, but we found simple averaging of estimates across observation periods to produce a higher value of inter-rater reliability due to over-weighting observation periods with fewest interactions.

**Conclusions:**

We recommend that researchers evaluating the inter-rater reliability of the QuIS by observing staff-inpatient interactions during observation periods representing the variety of ward conditions in which care takes place, should summarise inter-rater reliability by κ_*w*_, weighted according to our scheme A4. Observation period specific estimates should be combined into an overall, single summary statistic ${\widehat{\upkappa}}_{w\  random}$, using a random effects approach, with ${\widehat{\upkappa}}_{w\  random}$, to be interpreted as the mean of the distribution of κ_*w*_ across the variety of ward conditions. We draw attention to issues in the analysis and interpretation of inter-rater reliability studies incorporating distinct phases of data collection that may generalise more widely.

**Electronic supplementary material:**

The online version of this article (doi:10.1186/s12874-016-0266-4) contains supplementary material, which is available to authorized users.

## Background

The Quality of Interactions Schedule (QuIS) has its origin in observational research undertaken in 1989 by Clark & Bowling [1] in which the social content of interactions between patients and staff in nursing homes and long term stay wards for older people was rated to be positive, negative or neutral. The rating specifically relates to the social or conversational aspects of an interaction, such as the degree to which staff acknowledge the patient as a person, not to the adequacy of any care delivered during the interaction. Dean et al. [2] extended the rating by introducing distinctions within the positive and negative ratings, creating a five category scale as set out in Table 1. QuIS is now generally regarded as an ordinal scale ranging from the highest ranking, positive social interactions to the lowest ranking, negative restrictive interactions [3].

**Table 1:** Definitions of QuIS categories [2]

CATEGORY	Explanation
Positive social (+s)	Interaction principally involving ‘good, constructive, beneficial’ conversation and companionship.
Positive Care (+c)	Interactions during the appropriate delivery of physical care.
Neutral (N)	Brief, indifferent interactions not meeting the definitions of the other categories.
Negative protective (−p)	Providing care, keeping safe or removing from danger, but in a restrictive manner, without explanation or reassurance: in a way which disregards dignity or fails to demonstrate respect for the individual.
Negative restrictive (−r)	Interactions that oppose or resist peoples’ freedom of action without good reason, or which ignore them as a person.

Barker et al. [4] in a feasibility study of an intervention designed to improve the compassionate/social aspects of care experienced by older people in acute hospital wards, proposed the use of the QuIS as a direct assessment of this aspect of the quality of care received. This is a different context to that for which the QuIS was originally developed and extended, and it may well perform differently: wards may be busier and more crowded, beds may be curtained off, raters may have to position themselves more or less favourably in relation to the patients they are observing. A component of the feasibility work evaluated the suitability of the QuIS in the context of acute wards, and in particular its inter-rater-reliability [5]. Because of the lack of alternative assessments of quality of care it is likely that the QuIS will be used more widely, and any such use should be preceded by studies examining its suitability and its inter-rater reliability.

In this paper we describe the analysis of data from an inter-rater reliability study of the QuIS reported by McLean et al. [5]. Eighteen pairs of observers rated staff-inpatient interactions during two hour long observation periods purposively chosen to reflect the wide variety of conditions in which care is delivered in the hospital setting. The study should thus have captured differences in the quality of care across conditions, for example when staff were more or less busy. It is possible that inter-rater reliability could also vary depending on the same factors, and thus an overall statement of typical inter-rater reliability should reflect variability across observation periods in addition to sampling variability. We aim to establish a protocol for summarising data from inter-rater reliability studies of the QuIS, to facilitate consistency across future evaluations of its measurement properties. We summarise inter-rater reliability using kappa (κ) which quantifies the extent to which two raters agree in their ratings, over and above the agreement expected through chance alone. This is the most frequently used presentation of inter-rater reliability in applied health research, and is thus familiar to researchers in the area. When κ is calculated all differences in ratings are treated equally. Varying severity of disagreement between raters depending on the categories concerned can be accommodated in weighted κ, κ_*w*_, however standard weighting schemes give equal weight to disagreements an equal number of categories apart regardless of their position on the scale, and are thus not ideal for the QuIS. For example, a disagreement between the two adjacent positive categories is not equivalent to a disagreement between the adjacent positive care and neutral categories. Thus we aim to establish a set of weights to be used in κ_*w*_, that reflects the severity of misclassification between each pair of QuIS categories. We propose using meta-analytic techniques to combine the estimates of κ_*w*_ from the different observation periods to produce a single overall estimate of κ_*w*_.

## Methods

### QuIS observation

Following the training described by McLean et al. [5], each of 18 pairs of research staff observed, and QuIS rated all interactions involving either of two selected patients, during a two-hour long observation period. The 18 observation periods were selected with the intention of capturing a wide variety of conditions in which care is delivered to patients in acute wards, as this was the target of the intervention to be evaluated in a subsequent main trial. Observation was restricted to a single, large teaching hospital on the South Coast of England and took place in three wards, on weekdays, and at varying times of day between 8 am to 6 pm, including some periods when staff were expected to be busy (mornings) and others when staff might be less so.

The analysis of inter-rater reliability was restricted to staff-patient interactions rated by both raters, indicated by them reporting an interaction starting at the same time: interactions rated by only one rater were excluded. The percentage of interactions missed by either rater is reported, as is the Intra-class Correlation Coefficient (ICC) of total number of interactions reported by each rater in the observation periods.

### κ estimates of inter-rater reliability

Inter-rater agreement was assessed as Cohen’s κ [6] calculated from the cross-tabulation of ratings into the *k* = 5 QuIS categories of the interactions observed by both raters:

1 $$ \widehat{\upkappa}=\frac{p_o - {p}_{e\ }}{1-{p}_{e\ }}, $$

with *p*_*o*_ being the proportion of interactions with identical QuIS ratings and *p*_*e*_ being the proportion of interactions expected to be identical (∑_*i* = 1_^*k*^ *p*_*i. ﻿*_ *p*_.*i*_) calculated from the marginal proportions *p*_*i. *_ and *p*_*.i*_ of the cross-tabulation.

In the above, raters are only deemed to agree in their rating of an interaction if they record an identical QuIS category, and thus any ratings one point apart (for example ratings of + social and + care) are treated as disagreeing to the same extent as ratings a further distance apart (for example ratings of + social and - restrictive). To better reflect the severity of misclassification between pairs of QuIS categories weighted κ_*w*_ can be estimated as follows:

2 $$ {\widehat{\upkappa}}_w=\frac{p_{o\ (w)}-{p}_{e\ (w)}}{1-{p}_{e\ (w)}}, $$

where *p*_*o* (*w*)_ is the proportion of participants observed to agree according to a set of weights *w*_*ij*_

3 $$ {p}_{o\ (w)}={\displaystyle {\sum}_{i=1}^k{\displaystyle {\sum}_{j=1}^k{w}_{ij}{p}_{ij}}}, $$

and *p*_*e* (*w*)_ is the proportion of participants expected to agree according to the weights

4 $$ {p}_{e\ (w)}={\displaystyle {\sum}_{i=1}^k{\displaystyle {\sum}_{j=1}^k{w}_{ij}{p}_{i.}\kern0.2em {p}_{.j}}}. $$

In (3) *p*_*ij*_, for *i* and *j* = 1 … *k*, is the proportion of interactions rated as category *i* by the first rater and category *j* by the second. A weight *w*_*ij*_ is assigned to each combination restricted to lie in the interval 0 ≤ *w*_*ij*_ ≤ 1. Categories *i* and *j*, *i* ≠ *j* with *w*_*ij*_ = 1, indicate a pair of ratings deemed to reflect perfect agreement between the two raters. Only if *w*_*ij*_ is set at zero, *w*_*ij*_ = 0, are the ratings deemed to indicate complete disagreement. If 0 < *w*_*ij*_ < 1 for *i* ≠ *j*, ratings of *i* and *j* indicate ratings deemed to agree to the extent indicated by *w*_*ij*_. The precision of estimated κ_*w*_ from a sample of size *n* is indicated by the Wald 100(1- *α*)% confidence interval (CI):

5 $$ {\widehat{\upkappa}}_w-{z}_{\alpha /2}\times SE\left({\widehat{\upkappa}}_w\right)\le {\widehat{\upkappa}}_w\le {\widehat{\upkappa}}_w+{z}_{\alpha /2}\times SE\left({\widehat{\upkappa}}_w\right). $$

Fleiss et al. ([6], section 13.1) give an estimate of the standard error of ${\widehat{\upkappa}}_w$ as:

6 $$ \widehat{SE}\left({\widehat{\upkappa}}_w\right)=\frac{1}{\left(1-{p}_{e(w)}\right)\sqrt{n}}\sqrt{{\displaystyle {\sum}_{i=1}^k{\displaystyle {\sum}_{j=1}^k{p}_{i.}{p}_{.j}}}{\left[{w}_{ij}-\left({\overline{w}}_{i.}+{\overline{w}}_{.j}\right)\right]}^2-{p}_{e(w)}^2}, $$

where ${\overline{w}}_{i.}={\displaystyle {\sum}_{j=1}^k{p}_{.j}{w}_{ij}}$ and ${\overline{w}}_{.j}={\displaystyle {\sum}_{i=1}^k{p}_{i.}{w}_{ij}}$. Unweighted κ

is a special case.

We examined the sensitivity of ${\widehat{\upkappa}}_w$ to the choice of weighting scheme. Firstly we considered two standard schemes (linear and quadratic) described by Fleiss et al. [6] and implemented in Stata. Linear weighting deems the severity of disagreement between raters by one point to be the same at each point on the scale, and the weighting for disagreement by more than one point is the weight for a one-point disagreement multiplied by the number of categories apart. In quadratic weighting, disagreements two or more points apart are not simple multiples of the one-point weighting, but are still invariant to position on the scale. We believe that the severity of disagreement between two QuIS ratings a given number of categories apart, does depend on their position on the scale. The weighting schemes we devised as better reflections of misclassification between QuIS categories are described in Table 2. In weighting schemes A1 to A6 the severity of disagreements between each positive category and neutral, and each negative category and neutral was weighted to be 0.5; disagreement within the two positive categories was considered to be as severe as that within the two negative categories; and we considered a range of levels of weights (0.5 to 0.9) to reflect this. In schemes B1 to B3 disagreements between each positive category and neutral, and between each negative category and neutral were considered to be equally severe, but were given weight less than 0.5 (0.33, 0.25 and 0.00 respectively); severity of disagreement within the two positive categories was considered to be the same as that within the two negative categories. While in weighting schemes C1-C3, disagreement between the two positive categories (+social and + care) was considered to be less severe than that between the two negative categories (−protective and -restrictive).

**Table 2:** Weighting schemes

Weighting scheme		+ s	+ c	N	- p	- r	COMMENTS
Unweighted	+ social	1					Ignores the degree of misclassification between categories
	+ care	0	1				
	Neutral	0	0	1			
	- protective	0	0	0	1		
	- restrictive	0	0	0	0	1	
Linear	+ social	1					Standard weights 1 for ordinal variables in Stata. Weights 1-|i-j|/(k-1), where i and j index the rows and columns, and k the number of categories
	+ care	1	1				
	Neutral	0.5	0.5	1			
	- protective	0	0	0.5	1		
	- restrictive	0	0	0.5	1	1	
Quadratic	+ social	1					Standard weights 2 for ordinal variables in Stata. Weights 1 - {(i-j)/(k-1)}^2^.
	+ care	0.75	1				
	Neutral	0.5	0.75	1			
	- protective	0.25	0.5	0.75	1		
	- restrictive	0	0.25	0.5	0.75	1	
A: Weights given to neutral compared to a positive or negative = 0.5, assuming that misclassification between the positives is equal to misclassification between the negatives.							
Weighted A1	+ social	1					All possibilities from weighting misclassification between the two positives and the two negatives as 1 (will be the same as having only three categories, positive neutral and negative) to weighting it as 0.6. Weighting scheme 4 has a weights of 0.75 (half way between .5 and 1)
	+ care	1	1				
	Neutral	0.5	0.5	1			
	- protective	0	0	0.5	1		
	- restrictive	0	0	0.5	1	1	
Weighted A2	+ social	1					
	+ care	0.9	1				
	Neutral	0.5	0.5	1			
	- protective	0	0	0.5	1		
	- restrictive	0	0	0.5	0.9	1	
Weighted A3	+ social	1					
	+ care	0.8	1				
	Neutral	0.5	0.5	1			
	- protective	0	0	0.5	1		
	- restrictive	0	0	0.5	0.8	1	
Weighted A4	+ social	1					
	+ care	0.75	1				
	Neutral	0.5	0.5	1			
	- protective	0	0	0.5	1		
	- restrictive	0	0	0.5	0.75	1	
Weighted A5	+ social	1					
	+ care	0.7	1				
	Neutral	0.5	0.5	1			
	- protective	0	0	0.5	1		
	- restrictive	0	0	0.5	0.7	1	
Weighted A6	+ social	1					
	+ care	0.6	1				
	Neutral	0.5	0.5	1			
	- protective	0	0	0.5	1		
	- restrictive	0	0	0.5	0.6	1	
Weighting scheme		+ s	+ c	N	- p	- r	COMMENTS
B: Weights using less than 0.5 for neutral compared to a positive or negative and assuming that misclassification between the two positives is equal to misclassification between the two negatives							
Weighted B1	+ social	1					
	+ care	0.66	1				
	Neutral	0.33	0.33	1			
	- protective	0	0	0.33	1		
	- restrictive	0	0	0.33	0.66	1	
Weighted B2	+ social	1					
	+ care	0.5	1				
	Neutral	0.25	0.25	1			
	- protective	0	0	0.25	1		
	- restrictive	0	0	0.25	0.5	1	
Weighted B3	+ social	1					
	+ care	0.5	1				
	Neutral	0	0	1			
	- protective	0	0	0	1		
	- restrictive	0	0	0	0.5	1	
C: Weights assuming that misclassification between the two negative categories is less important than misclassification between the two positives and varying the neutral weights							
Weighted C1	+ social	1					
	+ care	0.5	1				
	Neutral	0.25	0.25	1			
	- protective	0	0	0.25	1		
	- restrictive	0	0	0.25	0.75	1	
Weighted C2	+ social	1					
	+ care	0.6	1				
	Neutral	0.4	0.4	1			
	- protective	0	0	0.4	1		
	- restrictive	0	0	0.4	0.8	1	
Weighted C3	+ social	1					
	+ care	0.66	1				
	Neutral	0.5	0.5	1			
	- protective	0	0	0.5	1		
	- restrictive	0	0	0.5	0.83	1	

Weighting scheme A4 is proposed as a good representation of the severity of disagreements between raters based on the judgement of the clinical authors (CMcL, PG and JB) for the following reasons:

i) There is an order between categories + social > +care > neutral > −protective > −restrictive
ii) Misclassification between any positive and any negative category is absolute and should not be considered to reflect any degree of agreement
iii) The most important misclassifications are between positive (combined), neutral and negative (combined) categories
iv) There is a degree of similarity between neutral and the two positive categories, and between neutral and the two negative categories
v) Misclassification *within* positive and negative categories do matter, but to a lesser extent

### Variation in ${\widehat{\upkappa}}_w$ over observation periods

We examined Spearman’s correlation between A4 weighted ${\widehat{\upkappa}}_w$ and time of day, interactions/patient hour, mean length of interactions and percentage of interactions less than one minute. ANOVA and two sample t-tests were used to examine differences in A4 weighted ${\widehat{\upkappa}}_w$ between wards and between mornings and afternoons.

### Overall ${\widehat{\upkappa}}_w$ combined over observation periods

To combine *g* (≥2) independent estimates of κ_*w*_, we firstly considered the naive approach of collapsing over observation periods to form a single cross-tabulation containing all the pairs of QuIS ratings, shown in Table 3a). An estimate, ${\widehat{\upkappa}}_{w\kern0.5em  collapsed}$, and its 95% CI, can be obtained from formulae (2) and (6).

**Table 3:** Cross-tabulation of QuIS ratings collapsed over all observation periods, and for the observation periods with lowest and highest unweighted κ

		Rater 2						Unweighted κ
		+ s	+ c	N	- p	- r	Total	
a) Collapsed table from all observation periods								
Rater 1	+ social	36	23	0	0	0	59 (17%)	0.55
	+ care	22	164	10	4	1	201 (57%)	
	Neutral	3	13	47	2	5	70 (20%)	
	- protective	0	5	2	7	0	14 (4%)	
	- restrictive	3	1	0	0	6	10 (3%)	
	Total	64 (18%)	206 (58%)	59 (17%)	13 (4%)	12 (3%)	354 (100%)	
b) Observation period with lowest unweighted κ								
Rater 1	+ social	2	4	0	0	0	6	0.30
	+ care	1	9	2	0	1	13	
	Neutral	0	2	2	1	0	5	
	- protective	0	0	0	0	0	0	
	- restrictive	0	0	0	0	1	1	
	Total	3	15	4	1	2	25	
c) Observation period with highest unweighted κ								
Rater 1	+ social	1	0	0	0	0	1	0.90
	+ care	0	11	0	0	0	11	
	Neutral	0	0	6	0	0	6	
	- protective	0	0	1	0	0	1	
	- restrictive	0	0	0	0	0	0	
	Total	1	11	7	0	0	19	

We next considered combining the *g* observation period specific estimates of κ_*w*_ using meta-analytic techniques. Firstly, using a fixed effects approach, the estimate ${\widehat{\upkappa}}_{wm}={\upkappa}_w+{\varepsilon}_m$ in the *m* ^*th*^ observation period is modelled as comprising the true underlying value of κ_*w*_ plus a component, *ε*_*m*_, reflecting sampling variability dependent on the number of interactions observed within the *m* ^*th*^ period: where κ_*w*_ is the common overall value, and *ε*_*m*_ is normally distributed with zero mean and variance ${V}_{wm} = SE{\left({\widehat{\upkappa}}_{wm}\right)}^2$. The inverse-variance estimate of κ_*w*_, based on the fixed effects model, ${\widehat{\upkappa}}_{w\kern0.5em  fixed}$, is a weighted combination of the estimates from each observation period:

7 $$ {\widehat{\upkappa}}_{w\  fixed}=\frac{{\displaystyle {\sum}_{m=1}^g}{\omega}_m\times {\widehat{\upkappa}}_{wm}}{{\displaystyle {\sum}_{m=1}^g}{\omega}_m}, $$

with meta-analytic weights, *ω*_*m*_, given by:

8 $$ {\omega}_m=\frac{1}{V_{wm}}. $$

Since study specific variances are not known, estimates ${\widehat{\omega}}_m$ with variance estimates ${\widehat{V}}_{wm} = \widehat{SE}{\left({\widehat{\upkappa}}_{wm}\ \right)}^2$ calculated from formula (6) for each of the *m* periods are used. The standard error of ${\widehat{\upkappa}}_{w\  fixed}$ is then:

9 $$ SE\left({\widehat{\upkappa}}_{w\  fixed}\right)=\sqrt{\frac{1}{{\displaystyle {\sum}_{m=1}^g}{\widehat{\omega}}_m}} $$

from which a 100(1- *α*)% CI for ${\widehat{\upkappa}}_{w\  fixed}$ can be obtained. ${\widehat{\upkappa}}_{w\  fixed}$ is the estimate ${\widehat{\upkappa}}_{w\  overall}$ combined over strata given by Fleiss et al. [6], here combining weighted ${\widehat{\upkappa}}_{wm}$ rather than unweighted ${\widehat{\upkappa}}_m$.

Equality of the *g* underlying, observation period specific values of κ_*w*_, is tested using a *χ* ^2^ test for heterogeneity:

10 $$ {\chi^2}_{heterogeneity}={\displaystyle {\sum}_{m=1}^g{\omega}_m}\times {\left({\widehat{\upkappa}}_{wm}-{\widehat{\upkappa}}_{w\  fixed}\right)}^2 $$

to be referred to *χ* ^2^ tables with *g* − 1 degrees of freedom. The hypothesis of equality in the *g* κ_*wm*_s is typically rejected if *χ* ^2^_*heterogeneity*_ lies above the *χ*_*g* − 1_^2^(0.95) percentile.

The fixed effects model assumes that all observation periods share a common value, κ_*w*_, with any differences in the observation period specific ${\widehat{\upkappa}}_{wm}$ being due to sampling error. Because of our expectation that inter-rater reliability will vary depending on ward characteristics and other aspects of specific periods of observation, our preference is for a more flexible model incorporating underlying variation in true κ_*wm*_ over the *m* periods within a random effects meta-analysis. The random effects model has ${\widehat{\upkappa}}_{wm}={\upkappa}_w+{\delta}_m+{\varepsilon}_m$, where *δ*_*m*_ is an observation period effect, independent of sampling error (the *ε*_*m*_ terms defined as for the fixed effects model). Variability in observed ${\widehat{\upkappa}}_{wm}$ about their underlying mean, κ_*w*_, is thus partitioned into a source of variation due to observation period characteristics captured by the *δ*_*m*_ terms, which are assumed to follow a Normal distribution: *δ*_*m*_ ~ *N*(0, *τ* ^2^), with *τ* ^2^ the variance in κ_*wm*_ across observation periods, and sampling variability. The inverse-variance estimate of κ_*w*_ for this model is:

11 $$ {\widehat{\upkappa}}_{w\  random}=\frac{{\displaystyle {\sum}_{m=1}^g}{\varOmega}_m\times {\widehat{\upkappa}}_{wm}}{{\displaystyle {\sum}_{m=1}^g}{\varOmega}_m}, $$

with meta-analytic weights, *Ω*_*m*_, given by:

12 $$ {\varOmega}_m=\frac{1}{V_{wm}+{\tau}^2}. $$

Observation period specific variance estimates ${\widehat{V}}_{wm}$ are used, and *τ* ^2^ also has to be estimated. A common choice is the Dersimonian-Laird estimator [7] defined as:

13 $$ {\widehat{\tau}}^2=\frac{{\chi^2}_{heterogeneity}-\left(g-1\right)}{{\displaystyle {\sum}_{m=1}^g}{\omega}_m-\left({\displaystyle {\sum}_{m=1}^g}{\upomega}_m^2\right)/\left({\displaystyle {\sum}_{m=1}^g}{\upomega}_m\right)} $$

usually truncated at 0 if the observed *χ* ^2^_*heterogeneity*_ < (*g* − 1). The estimate ${\widehat{\upkappa}}_{w\  random}$ is then:

14 $$ {\widehat{\upkappa}}_{w\  random}=\frac{{\displaystyle {\sum}_{m=1}^g}{\widehat{\varOmega}}_m\times {\widehat{\upkappa}}_{wm}}{{\displaystyle {\sum}_{m=1}^g}{\widehat{\varOmega}}_m}, $$

with

15 $$ {\widehat{\varOmega}}_m=\frac{1}{{\widehat{V}}_{wm}+{\widehat{\tau}}^2}, $$

and an estimate of the standard error of ${\widehat{\upkappa}}_{w\  random}$ is:

16 $$ \widehat{SE}\left({\widehat{\upkappa}}_{w\  random}\right)=\sqrt{\frac{1}{{\displaystyle {\sum}_{m=1}^g}{\widehat{\varOmega}}_m}} $$

leading to 100(1- *α*)% CIs for ${\widehat{\upkappa}}_{w\  random}$.

The role of *τ* ^2^ is that of a tuning parameter: When *τ* ^2^ = 0 there is no variation in the underlying *κ*_*w*_, and the fixed effects estimate, ${\widehat{\upkappa}}_{w\  fixed}$ is obtained. At the other extreme, as *τ* ^2^ becomes larger, the ${\widehat{\varOmega}}_m$ become close to constant, so that each observation period is equally weighted and ${\widehat{\upkappa}}_{w\  random}$ becomes the simple average of observation period specific estimates:

17 $$ {\widehat{\upkappa}}_{w\  averaged}\kern0.75em =\frac{{\displaystyle {\sum}_{m=1}^g}{\widehat{\upkappa}}_{wm}}{g}. $$

${\widehat{\upkappa}}_{w\  averaged}$ ignores the impact of number of interactions on the precision of the observation period specific estimates. The standard error for ${\widehat{\upkappa}}_{w\  averaged}$ is estimated by:

18 $$ \widehat{SE}\left({\widehat{\upkappa}}_{w\  averaged}\right)=\sqrt{\frac{{\displaystyle {\sum}_{m=1}^g}{\widehat{V}}_{wm}}{g^2}}. $$

### Obtaining estimates of ${\widehat{\upkappa}}_w$ from Stata

The inverse-variance fixed and random effects estimates can be obtained from command metan [8] in Stata by feeding in pre-calculated effect estimates (variable X1) and their standard errors (variable X2). When X1 contains the *g* estimates of ${\widehat{\upkappa}}_{wm}$, X2 their standard errors $\sqrt{\ {\widehat{V}}_{wm}}$, and variable OPERIOD (labelled “Observation Period”) an indicator of observation periods, inverse-variance estimates are obtained from the command:

### metan X1 X2, second (random) lcols (OPERIOD) xlab(0, 0.2, 0.4, 0.6, 0.8, 1) effect(X1)

The “second(random)” option requests the ${\widehat{\upkappa}}_{w\  random}$ estimate in addition to ${\widehat{\upkappa}}_{w\  fixed}$. The “lcols” and “xlab” options control the appearance of the Forest plot of observation specific estimates, combined estimates, and their 95% CIs.

## Results

Across the 18 observation periods 447 interactions were observed, of which 354 (79%) were witnessed by both raters and form the dataset from which inter-rater reliability was estimated. The ICC for the total number of interactions recorded by each rater for the same observation period was high (ICC = 0.97: 95%CI: 0.92 to 0.99, *n* = 18). The occasional absence of patients from ward areas for short periods of time resulted in interactions being recorded for 67 patient hours (compared to the planned 72 h). The mean rate of interactions was 6.7 interactions/patient/hour. More detailed results are given by McLean et al. [5].

In Table 3a) the cross-tabulation of ratings by the two raters can be seen collapsed over the 18 observation periods. Two specific observation periods are also shown: in 3b) the period demonstrating lowest unweighted $\widehat{\upkappa}$ ($\widehat{\upkappa}$ =0.30); and in 3c) the period demonstrating highest unweighted $\widehat{\upkappa}$ ($\widehat{\upkappa}$ =0.90). From 3a) it can be seen that the majority of interactions are rated to be positive, between 17% and 20% are rated to be neutral, and 7% as negative (from the margins of the table), and this imbalance in the marginal frequencies would be expected to reduce chance adjusted κ.

**Fig. 1:**
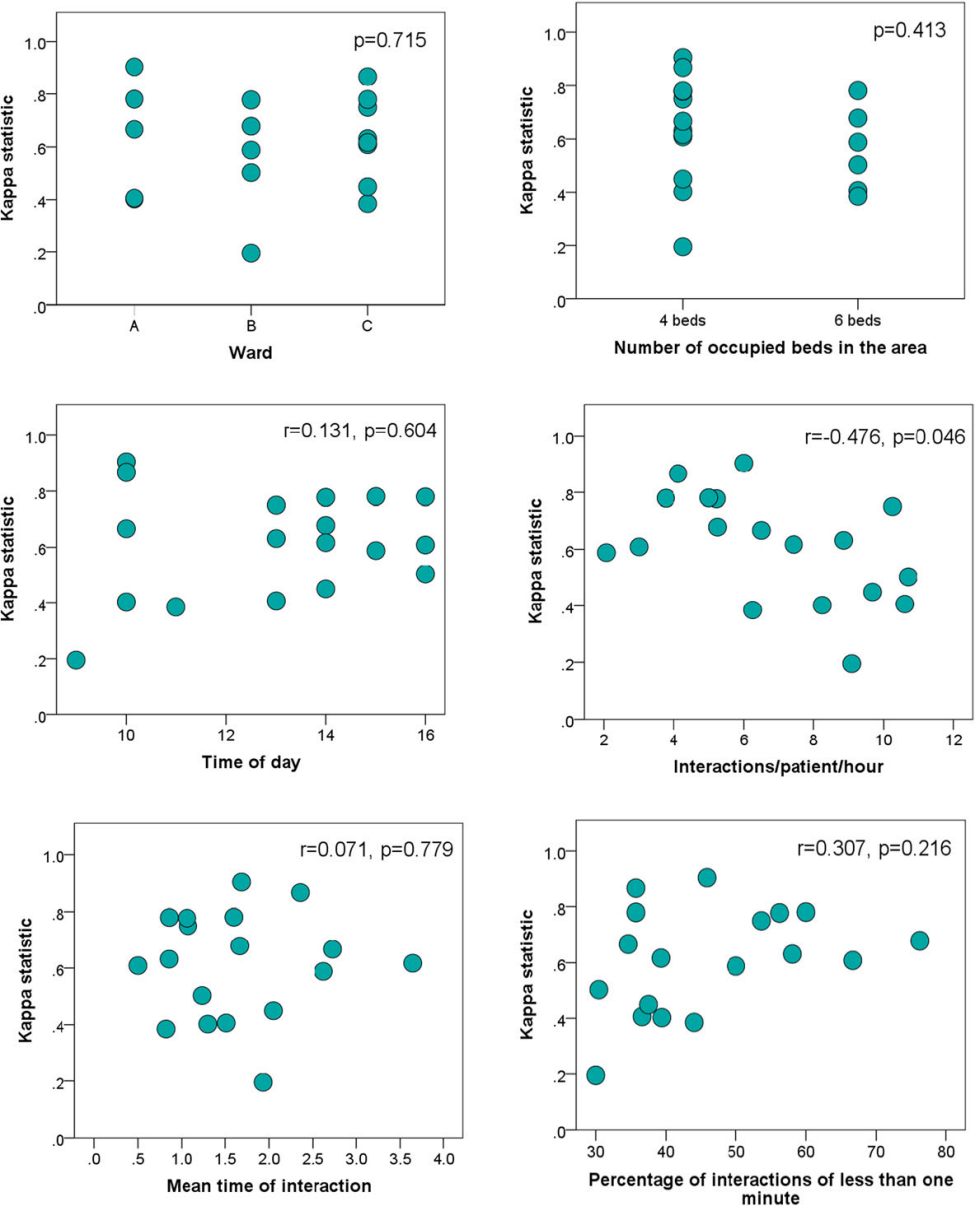
Variability of A4 weighted ${\widehat{\upkappa}}_{wm}$ in relation to observation period characteristics (*n* = 18). P values relate to Spearman’s correlation

Scatterplots of A4 weighted ${\widehat{\upkappa}}_{wm}$ against observation period characteristics are shown in Fig. 1. One of the characteristics (interactions/patient/hour) was sufficiently associated with A4 weighted ${\widehat{\upkappa}}_{wm}$ to achieve statistical significance (*P* = 0.046).

In Table 4 it can be seen that the various combined estimates of κ_*w*_ did not vary greatly depending on the method of meta-analysis or on the choice of weighting scheme. However, there was greater variability in *χ* ^2^_*heterogeneity*_. For all weighting schemes except unweighted, B2, B3, and C1, there was statistically significant heterogeneity by virtue of *χ* ^2^_*heterogeneity*_ exceeding the *χ*_17_^2^(0.95) cut-point of 27.59.

**Table 4:** Combined estimates of κ_*w*_ with different weighting schemes

Weighting scheme	${\widehat{\upkappa}}_{w\ collapsed}$ (95% CI)	${\widehat{\upkappa}}_{w\ fixed}$ (95% CI)	*χ* ^2^_*heterogeneity*_	${\widehat{\upkappa}}_{w\ random}$ (95% CI)	${\widehat{\upkappa}}_{w\ averaged}$ (95% CI)
Unweighted	0.55 (0.49, 0.62)	0.52 (0.45, 0.59)	21.20	0.53 (0.45, 0.60)	0.57 (0.48, 0.65)
Linear	0.58 (0.51, 0.65)	0.52 (0.45, 0.59)	35.67	0.56 (0.46, 0.66)	0.59 (0.51, 0.68)
Quadratic	0.61 (0.50, 0.71)	0.53 (0.44, 0.62)	38.71	0.59 (0.45, 0.74)	0.63 (0.52, 0.73)
A1	0.64 (0.56, 0.73)	0.51 (0.43, 0.59)	47.15	0.62 (0.48, 0.77)	0.66 (0.57, 0.75)
A2	0.62 (0.54, 0.70)	0.50 (0.43, 0.57)	45.75	0.60 (0.47, 0.73)	0.64 (0.54, 0.73)
A3	0.60 (0.53, 0.68)	0.51 (0.44, 0.58)	39.28	0.58 (0.47, 0.69)	0.62 (0.53, 0.71)
A4	0.60 (0.53, 0.67)	0.51 (0.44, 0.58)	36.04	0.57 (0.47, 0.68)	0.61 (0.52, 0.70)
A5	0.59 (0.52, 0.66)	0.52 (0.45, 0.59)	33.22	0.56 (0.46, 0.67)	0.60 (0.52, 0.69)
A6	0.58 (0.51, 0.64)	0.52 (0.45, 0.59)	29.10	0.55 (0.46, 0.64)	0.59 (0.51, 0.67)
B1	0.59 (0.53, 0.66)	0.53 (0.46, 0.59)	30.52	0.56 (0.47, 0.66)	0.60 (0.52, 0.69)
B2	0.58 (0.51, 0.65)	0.53 (0.46, 0.59)	26.01	0.55 (0.46, 0.64)	0.59 (0.51, 0.67)
B3	0.59 (0.53, 0.66)	0.53 (0.47, 0.60)	25.11	0.55 (0.47, 0.64)	0.60 (0.51, 0.68)
C1	0.58 (0.51, 0.65)	0.53 (0.46, 0.59)	26.05	0.55 (0.46, 0.64)	0.59 (0.51, 0.67)
C2	0.58 (0.51, 0.65)	0.52 (0.45, 0.59)	28.82	0.55 (0.46, 0.65)	0.60 (0.51, 0.68)
C3	0.58 (0.51, 0.65)	0.52 (0.45, 0.59)	31.26	0.56 (0.46, 0.66)	0.60 (0.51, 0.68)
min-max ${\widehat{\upkappa}}_w$ across weighting schemes	0.55–0.64	0.50–0.53	*χ*_17_^2^(0.95) =27.59	0.53–0.62	0.57–0.66

**Fig. 2:**
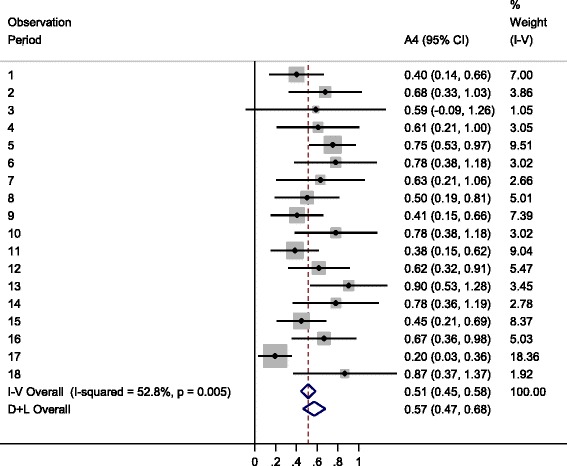
Forest plot showing observation period specific A4 weighted ${\widehat{\upkappa}}_{wm}$, ${\widehat{\upkappa}}_{w\  fixed}$, and ${\widehat{\upkappa}}_{w\  random}$

Figure 2 shows the Forest plot demonstrating the variability in ${\widehat{\upkappa}}_{wm}$ over observation periods, ${\widehat{\upkappa}}_{w\  fixed}$, and ${\widehat{\upkappa}}_{w\  random}$, for the A4 weighting scheme. Estimate ${\widehat{\upkappa}}_{w\  fixed}$ and its 95% CI is shown below observation specific estimates to the right of the plot, on the line labelled “I-V Overall”. The line below labelled “D+L Overall” presents ${\widehat{\upkappa}}_{w\  random}$ and its 95% CI. Both estimates are identical to those shown in Table 4. The final column “% Weight (I-V)” relates to the meta-analytic weights, ${\widehat{\omega}}_m$, not the A4 weighting scheme adopted for κ_*w*_.

## Discussion

We consider the most appropriate estimate of inter-rater reliability of the QuIS to be 0.57 (95% CI 0.47 to 0.68) indicative of only moderate inter-rater reliability. The finding was not unexpected, the QuIS categories can be difficult to distinguish and though positioned as closely together as possible, the two raters had different lines of view, potentially impacting on their QuIS ratings. The estimate of inter-rater reliability is based on our A4 weighting scheme with observation specific estimates combined using random effects meta-analysis. Combined estimates of κ_*w*_ were not overly sensitive to the choice of weighting scheme amongst those we considered as plausible representations of the severity of misclassification between QuIS categories. We recommend a random effects approach to combining observation period specific estimates, ${\widehat{\upkappa}}_{wm}$, to reflect the inherent variation anticipated over observation periods.

There are undoubtedly other weighting schemes that fulfil all the criteria on which we chose weighting scheme A4, but the evidence from our analyses suggests that it makes relatively little difference to the resultant ${\widehat{\upkappa}}_{w\  random}$. In the absence of any other basis for determining weights, our scheme A4 has the virtue of simplicity. A key issue is that researchers should not examine the ${\widehat{\upkappa}}_w$ resulting from a variety of weighting schemes, and then choose the scheme giving highest inter-rater reliability. The adoption of a standard set of weights also facilitates comparison of inter-rater reliability across different studies of QuIS.

We compared four approaches to estimating overall κ_w_. We do not recommend the simplest of these, ${\widehat{\upkappa}}_{w\  collapsed}$, based on estimating κ_*w*_ from the cross-tabulation of all ratings collapsed over observation periods: generally collapsing involves a risk of confounding by stratum effects. Comparing the remaining estimates it can be seen that ${\widehat{\upkappa}}_{w\  random}$ lies between the fixed effects, ${\widehat{\upkappa}}_{w\  fixed}$, and the averaged estimate, ${\widehat{\upkappa}}_{w\  averaged}$, for all the weighting schemes we considered. ${\widehat{\upkappa}}_{w\  averaged}$ gives equal meta-analytic weight to each observation period, and thus up-weights periods with highest variance compared to ${\widehat{\upkappa}}_{w\  fixed}$. The observation periods with highest variance are those with fewest interactions/patient/hour of observation, and it can be seen from Fig. 1 that these periods tend to have highest ${\widehat{\upkappa}}_{wm}$. A possible explanation being that with fewer interactions it is easier for observers to see and hear the interactions and thus make their QuIS ratings which would be anticipated to result in more accuracy and agreement. Thus ${\widehat{\upkappa}}_{w\  averaged}$ might be expected to over-estimate inter-rater reliability and should be avoided. We recommend a random, rather than fixed effects approach to combining because variation in κ_*wm*_ across observation periods was anticipated. Observation periods were chosen with the intention of representing the broad range of situations in which staff-inpatient interactions take place. At different times of day staff will be more or less busy, and this more or less guarantees heterogeneity in observation period specific inter-rater reliability.

Böhning et al. [9] identified several practical issues relating to inverse variance estimators in meta-analysis. For example and most importantly, that estimation is no longer unbiased when estimated rather than known variances are used in the meta-analytic weights. This bias is less extreme for larger sample sizes in each constituent study. We included 354 interactions across the 18 observation periods, on average about 20 per period, but it is not clear whether this is sufficient for meaningful bias to be eradicated. A further issue relates to possible misunderstanding of the single combined estimate as applying to all observation periods: a correct interpretation being that the single estimate relates to the mean of the distribution of κ_*wm*_ over observation periods. An alternative might be to present the range of values that κ_*w*_ is anticipated to take over most observation periods. This would be an unfamiliar presentation for most researchers.

Meta-analysis of $\widehat{\upkappa}$ over studies following a systematic review has been considered by Sun [10] where fixed and random effects approaches are described, but the latter adopting the Hedges [11], rather than the conventional Dersimonian-Laird estimate of *τ* ^2^. Alternatives to the DerSimonian-Laird estimator are available including the REML estimate, or the Hartung-Knapp-Sidik-Jonkman method [12]. Friede et al. [13] examine properties of the DerSimonian-Laird estimator when there are only two observation periods and conclude that in such circumstances other estimators are preferable: McLean et al’s study [5] was based on sufficient observation periods to make these problems unlikely. Sun addressed the issue of publication bias amongst inter-rater reliability studies found by searching the literature. Here we included data from all observation periods, irrespective of the estimate ${\widehat{\upkappa}}_{wm}$. Sun performed subgroup analyses of studies according to the degree of training of the raters involved, and also drew a distinction between inter-rater reliability studies where both raters can be considered to be equivalent and a study [14] comparing ratings from hospital nurses with those from an expert which would more appropriately have been analysed using sensitivity, specificity and related techniques. The QuIS observations were carried out by raters who had all received the training developed by McLean et al: though there was variation in experience of QuIS a further source of inter-rater unreliability relating to the different lines of view from each rater’s position was also considered to be important.

In the inter-rater study we describe, in some instances the same rater was involved in more than one observation period, and this potentially violates the assumption of independence across observation periods, which would be anticipated to lead to increased variance in an overall estimate, ${\widehat{\upkappa}}_w$. A random effects approach is more suitable in this regard as it catches some of the additional variance, coping with extra-dispersion whether it arises from unobserved heterogeneity or from correlation across observation periods.

Though we have considered analysis choices that need to be made when summarising information on the inter-rater reliability of the QuIS, the issues we address are relevant to inter-rater reliability studies more generally. Firstly, where weighted κ_*w*_ rather than unweighted κ is thought to be a better summary of differing degrees of disagreement between raters, it is important that the weighting scheme be decided in advance. Secondly, where a study comprises distinct subsets of data collection, the method of combining information needs to be considered. It is likely that data in larger inter-rater reliability studies would need to be collected in distinct phases, but the lack of attention to combining ${\widehat{\upkappa}}_m$ over subsets within a study suggests that researchers often ignore the issue, adopting the easiest approach of collapsing to obtain a single estimate of κ. We would advise taking account of structure in data collection by either a fixed or random effects meta-analysis approach, the latter being appropriate where variation across subsets is anticipated or plausible. Our example dataset illustrates a potential source of bias in the simple average of subset specific estimates, ${\widehat{\upkappa}}_m$. Finally, in the context of meta-analysis over studies, Sun considered the issue of bias arising from the selection of studies for publication. In the context of combining over subsets of data collection within a study, it is possible to imagine circumstances where authors might choose to omit selected subsets, but a good reason would have to be given to justify such a step and the omitted data described.

## Conclusions

Researchers using the QuIS to evaluate the quality of staff/inpatient interactions should check its suitability in new settings, and (possibly as part of staff training) its inter-rater reliability. In practice such studies are likely to follow a similar protocol to that adopted by McClean et al.: involving the multiple observers to be employed in a subsequent main study, over a variety of wards similar to those planned for the main study; and preferably taking place at different times of day. We recommend inter-rater reliability be estimated using our A4 weighting scheme and a random effects meta-analytic approach to combining estimates over observation periods, ${\widehat{\upkappa}}_{w\  random}$, be adopted. The ${\widehat{\upkappa}}_{w\  random}$ estimate should be presented with its 95% confidence interval reflecting precision of estimation achieved from the available number and length of observation periods.

## Additional file

Additional file 1: Table S1. Cross-classification of ratings for each of the 18 observation periods and period specific covariates^1^. (DOCX 36 kb)

## Abbreviations

κ: Unweighted kappa
κ_*w*_: Weighted kappa
ICC: Intra-cluster correlation
QuIS: Quality of Interaction Schedule
