# Supplementary material for: Inter-rater reliability of the QuIS as an assessment of the quality of staff-inpatient interactions
Source: BMC Med Res Methodol. 2016 Dec 7;16:171. doi: 10.1186/s12874-016-0266-4 (PMC5142422; doi:10.1186/s12874-016-0266-4)
Supplement: Additional file 1: Table S1. — Cross-classification of ratings for each of the 18 observation periods and period specific covariates1. (DOCX 36 kb) [file 12874_2016_266_MOESM1_ESM.docx]

**Supplementary table: cross-classification of ratings for each of the 18 observation periods and period specific covariates^1^.**

| **Observation period 1**  $\boldsymbol{x}_{\boldsymbol{1}}$**=A,** $\boldsymbol{x}_{\boldsymbol{2}}$**=4,** $\boldsymbol{x}_{\boldsymbol{3}}$**=10,** $\boldsymbol{x}_{\boldsymbol{4}}$**=8.25,** $\boldsymbol{x}_{\boldsymbol{5}}$**=1.30,** $\boldsymbol{x}_{\boldsymbol{6}}$**=39.4.** | | **Observer 2** | | | | | |
| --- | --- | --- | --- | --- | --- | --- | --- |
|  |  | **+ s** | **+ c** | **N** | **- p** | **- r** | **Total** |
| **Observer 1** | **+ social** | 1 | 1 | 0 | 0 | 0 | 2 |
|  | **+ care** | 3 | 17 | 1 | 0 | 0 | 21 |
|  | **Neutral** | 0 | 1 | 2 | 1 | 0 | 4 |
|  | **- protective** | 0 | 1 | 0 | 0 | 0 | 1 |
|  | **- restrictive** | 0 | 0 | 0 | 0 | 0 | 0 |
|  | **Total** | 4 | 20 | 3 | 1 | 0 | 28 |
| **Observation period 2**  $\boldsymbol{x}_{\boldsymbol{1}}$**=B,** $\boldsymbol{x}_{\boldsymbol{2}}$**=6,** $\boldsymbol{x}_{\boldsymbol{3}}$**=14,** $\boldsymbol{x}_{\boldsymbol{4}}$**=5.25,** $\boldsymbol{x}_{\boldsymbol{5}}$**=1.67,** $\boldsymbol{x}_{\boldsymbol{6}}$**=76.2.** | | **Observer 2** | | | | | |
|  |  | **+ s** | **+ c** | **N** | **- p** | **- r** | **Total** |
| **Observer 1** | **+ social** | 1 | 0 | 0 | 0 | 0 | 1 |
|  | **+ care** | 1 | 5 | 0 | 0 | 0 | 6 |
|  | **Neutral** | 1 | 1 | 2 | 0 | 0 | 4 |
|  | **- protective** | 0 | 0 | 0 | 3 | 0 | 3 |
|  | **- restrictive** | 0 | 1 | 0 | 0 | 0 | 1 |
|  | **Total** | 3 | 7 | 2 | 3 | 0 | 15 |
| **Observation period 3**  $\boldsymbol{x}_{\boldsymbol{1}}$**=B,** $\boldsymbol{x}_{\boldsymbol{2}}$**=6,**$\boldsymbol{x}_{\boldsymbol{3}}$**=15,** $\boldsymbol{x}_{\boldsymbol{4}}$**=2.07,** $\boldsymbol{x}_{\boldsymbol{5}}$**=2.63,** $\boldsymbol{x}_{\boldsymbol{6}}$**=50.** | | **Observer 2** | | | | | |
|  |  | **+ s** | **+ c** | **N** | **- p** | **- r** | **Total** |
| **Observer 1** | **+ social** | 1 | 0 | 0 | 0 | 0 | 1 |
|  | **+ care** | 1 | 5 | 0 | 0 | 0 | 6 |
|  | **Neutral** | 0 | 0 | 0 | 0 | 0 | 0 |
|  | **- protective** | 0 | 0 | 0 | 0 | 0 | 0 |
|  | **- restrictive** | 0 | 0 | 0 | 0 | 0 | 0 |
|  | **Total** | 2 | 5 | 0 | 0 | 0 | 7 |

**Supplementary table C^td^:**

| **Observation period 4**  $\boldsymbol{x}_{\boldsymbol{1}}$**=C,** $\boldsymbol{x}_{\boldsymbol{2}}$**=4,** $\boldsymbol{x}_{\boldsymbol{3}}$**=16,** $\boldsymbol{x}_{\boldsymbol{4}}$**=3,** $\boldsymbol{x}_{\boldsymbol{5}}$**=0.50,** $\boldsymbol{x}_{\boldsymbol{6}}$**=66.7.** | | **Observer 2** | | | | | |
| --- | --- | --- | --- | --- | --- | --- | --- |
|  |  | **+ s** | **+ c** | **N** | **- p** | **- r** | **Total** |
| **Observer 1** | **+ social** | 1 | 1 | 0 | 0 | 0 | 2 |
|  | **+ care** | 0 | 8 | 0 | 0 | 0 | 8 |
|  | **Neutral** | 0 | 1 | 1 | 0 | 0 | 2 |
|  | **- protective** | 0 | 0 | 0 | 0 | 0 | 0 |
|  | **- restrictive** | 0 | 0 | 0 | 0 | 0 | 0 |
|  | **Total** | 1 | 10 | 1 | 0 | 0 | 12 |
| **Observation period 5**  $\boldsymbol{x}_{\boldsymbol{1}}$**=C,** $\boldsymbol{x}_{\boldsymbol{2}}$**=4,** $\boldsymbol{x}_{\boldsymbol{3}}$**=13,** $\boldsymbol{x}_{\boldsymbol{4}}$**=10.25,** $\boldsymbol{x}_{\boldsymbol{5}}$**=1.07,** $\boldsymbol{x}_{\boldsymbol{6}}$**=53.7.** | | **Observer 2** | | | | | |
|  |  | **+ s** | **+ c** | **N** | **- p** | **- r** | **Total** |
| **Observer 1** | **+ social** | 2 | 2 | 0 | 0 | 0 | 4 |
|  | **+ care** | 6 | 17 | 0 | 0 | 0 | 23 |
|  | **Neutral** | 0 | 0 | 7 | 0 | 0 | 7 |
|  | **- protective** | 0 | 0 | 1 | 0 | 0 | 1 |
|  | **- restrictive** | 0 | 0 | 0 | 0 | 1 | 1 |
|  | **Total** | 8 | 19 | 8 | 0 | 1 | 36 |
| **Observation period 6**  $\boldsymbol{x}_{\boldsymbol{1}}$**=B,** $\boldsymbol{x}_{\boldsymbol{2}}$**=4,** $\boldsymbol{x}_{\boldsymbol{3}}$**=14,** $\boldsymbol{x}_{\boldsymbol{4}}$**=5.22,** $\boldsymbol{x}_{\boldsymbol{5}}$**=1.06,** $\boldsymbol{x}_{\boldsymbol{6}}$**=56.3.** | | **Observer 2** | | | | | |
|  |  | **+ s** | **+ c** | **N** | **- p** | **- r** | **Total** |
| **Observer 1** | **+ social** | 2 | 1 | 0 | 0 | 0 | 3 |
|  | **+ care** | 0 | 7 | 0 | 0 | 0 | 7 |
|  | **Neutral** | 0 | 1 | 3 | 0 | 0 | 4 |
|  | **- protective** | 0 | 0 | 0 | 0 | 0 | 0 |
|  | **- restrictive** | 0 | 0 | 0 | 0 | 0 | 0 |
|  | **Total** | 2 | 9 | 3 | 0 | 0 | 14 |

**Supplementary table C^td^:**

| **Observation period 7**  $\boldsymbol{x}_{\boldsymbol{1}}$**=C,** $\boldsymbol{x}_{\boldsymbol{2}}$**=4,** $\boldsymbol{x}_{\boldsymbol{3}}$**=13,** $\boldsymbol{x}_{\boldsymbol{4}}$**=8.86,** $\boldsymbol{x}_{\boldsymbol{5}}$**=0.85,** $\boldsymbol{x}_{\boldsymbol{6}}$**=58.1.** | | **Observer 2** | | | | | |
| --- | --- | --- | --- | --- | --- | --- | --- |
|  |  | **+ s** | **+ c** | **N** | **- p** | **- r** | **Total** |
| **Observer 1** | **+ social** | 0 | 0 | 0 | 0 | 0 | 0 |
|  | **+ care** | 0 | 14 | 1 | 0 | 0 | 15 |
|  | **Neutral** | 0 | 2 | 4 | 0 | 0 | 6 |
|  | **- protective** | 0 | 0 | 0 | 0 | 0 | 0 |
|  | **- restrictive** | 0 | 0 | 0 | 0 | 0 | 0 |
|  | **Total** | 0 | 16 | 5 | 0 | 0 | 21 |
| **Observation period 8**  $\boldsymbol{x}_{\boldsymbol{1}}$**=B,** $\boldsymbol{x}_{\boldsymbol{2}}$**=6,** $\boldsymbol{x}_{\boldsymbol{3}}$**=16,** $\boldsymbol{x}_{\boldsymbol{4}}$**=10.70,** $\boldsymbol{x}_{\boldsymbol{5}}$**=1.24,** $\boldsymbol{x}_{\boldsymbol{6}}$**=30.4.** | | **Observer 2** | | | | | |
|  |  | **+ s** | **+ c** | **N** | **- p** | **- r** | **Total** |
| **Observer 1** | **+ social** | 3 | 2 | 0 | 0 | 0 | 5 |
|  | **+ care** | 3 | 9 | 0 | 0 | 0 | 12 |
|  | **Neutral** | 0 | 0 | 0 | 0 | 0 | 0 |
|  | **- protective** | 0 | 1 | 0 | 1 | 0 | 2 |
|  | **- restrictive** | 0 | 0 | 0 | 0 | 0 | 0 |
|  | **Total** | 6 | 12 | 0 | 1 | 0 | 19 |
| **Observation period 9**  $\boldsymbol{x}_{\boldsymbol{1}}$**=A,** $\boldsymbol{x}_{\boldsymbol{2}}$**=6,** $\boldsymbol{x}_{\boldsymbol{3}}$**=13,** $\boldsymbol{x}_{\boldsymbol{4}}$**=10.60,** $\boldsymbol{x}_{\boldsymbol{5}}$**=1.51,** $\boldsymbol{x}_{\boldsymbol{6}}$**=36.6.** | | **Observer 2** | | | | | |
|  |  | **+ s** | **+ c** | **N** | **- p** | **- r** | **Total** |
| **Observer 1** | **+ social** | 2 | 4 | 0 | 0 | 0 | 6 |
|  | **+ care** | 1 | 9 | 2 | 0 | 1 | 13 |
|  | **Neutral** | 0 | 2 | 2 | 1 | 0 | 5 |
|  | **- protective** | 0 | 0 | 0 | 0 | 0 | 0 |
|  | **- restrictive** | 0 | 0 | 0 | 0 | 1 | 1 |
|  | **Total** | 3 | 15 | 4 | 1 | 2 | 25 |

**Supplementary table C^td^:**

| **Observation period 10**  $\boldsymbol{x}_{\boldsymbol{1}}$**=A,** $\boldsymbol{x}_{\boldsymbol{2}}$**=6,** $\boldsymbol{x}_{\boldsymbol{3}}$**=15,** $\boldsymbol{x}_{\boldsymbol{4}}$**=5,** $\boldsymbol{x}_{\boldsymbol{5}}$**=1.60,** $\boldsymbol{x}_{\boldsymbol{6}}$**=60.** | | **Observer 2** | | | | | |
| --- | --- | --- | --- | --- | --- | --- | --- |
|  |  | **+ s** | **+ c** | **N** | **- p** | **- r** | **Total** |
| **Observer 1** | **+ social** | 4 | 0 | 0 | 0 | 0 | 4 |
|  | **+ care** | 1 | 5 | 0 | 0 | 0 | 6 |
|  | **Neutral** | 0 | 0 | 3 | 0 | 0 | 3 |
|  | **- protective** | 0 | 0 | 0 | 0 | 0 | 0 |
|  | **- restrictive** | 0 | 0 | 0 | 0 | 0 | 0 |
|  | **Total** | 5 | 5 | 3 | 0 | 0 | 13 |
| **Observation period 11**  $\boldsymbol{x}_{\boldsymbol{1}}$**=C,** $\boldsymbol{x}_{\boldsymbol{2}}$**=6,** $\boldsymbol{x}_{\boldsymbol{3}}$**=11,** $\boldsymbol{x}_{\boldsymbol{4}}$**=6.25,** $\boldsymbol{x}_{\boldsymbol{5}}$**=0.82,** $\boldsymbol{x}_{\boldsymbol{6}}$**=44.** | | **Observer 2** | | | | | |
|  |  | **+ s** | **+ c** | **N** | **- p** | **- r** | **Total** |
| **Observer 1** | **+ social** | 0 | 0 | 0 | 0 | 0 | 0 |
|  | **+ care** | 1 | 6 | 2 | 3 | 0 | 12 |
|  | **Neutral** | 0 | 0 | 6 | 0 | 4 | 10 |
|  | **- protective** | 0 | 0 | 0 | 0 | 0 | 0 |
|  | **- restrictive** | 0 | 0 | 0 | 0 | 1 | 1 |
|  | **Total** | 1 | 6 | 8 | 3 | 5 | 23 |
| **Observation period 12**  $\boldsymbol{x}_{\boldsymbol{1}}$**=C,** $\boldsymbol{x}_{\boldsymbol{2}}$**=4,** $\boldsymbol{x}_{\boldsymbol{3}}$**=14,** $\boldsymbol{x}_{\boldsymbol{4}}$**=7.43,** $\boldsymbol{x}_{\boldsymbol{5}}$**=3.64,** $\boldsymbol{x}_{\boldsymbol{6}}$**=39.3.** | | **Observer 2** | | | | | |
|  |  | **+ s** | **+ c** | **N** | **- p** | **- r** | **Total** |
| **Observer 1** | **+ social** | 7 | 0 | 0 | 0 | 0 | 7 |
|  | **+ care** | 2 | 6 | 2 | 0 | 0 | 10 |
|  | **Neutral** | 0 | 1 | 0 | 0 | 1 | 2 |
|  | **- protective** | 0 | 1 | 0 | 0 | 0 | 1 |
|  | **- restrictive** | 0 | 0 | 0 | 0 | 3 | 3 |
|  | **Total** | 9 | 8 | 2 | 0 | 4 | 23 |

**Supplementary table C^td^:**

| **Observation period 13**  $\boldsymbol{x}_{\boldsymbol{1}}$**=A,** $\boldsymbol{x}_{\boldsymbol{2}}$**=4,** $\boldsymbol{x}_{\boldsymbol{3}}$**=10,** $\boldsymbol{x}_{\boldsymbol{4}}$**=6.00,** $\boldsymbol{x}_{\boldsymbol{5}}$**=1.69,** $\boldsymbol{x}_{\boldsymbol{6}}$**=45.8.** | | **Observer 2** | | | | | |
| --- | --- | --- | --- | --- | --- | --- | --- |
|  |  | **+ s** | **+ c** | **N** | **- p** | **- r** | **Total** |
| **Observer 1** | **+ social** | 1 | 0 | 0 | 0 | 0 | 1 |
|  | **+ care** | 0 | 11 | 0 | 0 | 0 | 11 |
|  | **Neutral** | 0 | 0 | 6 | 0 | 0 | 6 |
|  | **- protective** | 0 | 0 | 1 | 0 | 0 | 1 |
|  | **- restrictive** | 0 | 0 | 0 | 0 | 0 | 0 |
|  | **Total** | 1 | 11 | 7 | 0 | 0 | 19 |
| **Observation period 14**  $\boldsymbol{x}_{\boldsymbol{1}}$**=C,** $\boldsymbol{x}_{\boldsymbol{2}}$**=4,** $\boldsymbol{x}_{\boldsymbol{3}}$**=16,** $\boldsymbol{x}_{\boldsymbol{4}}$**=3.77,** $\boldsymbol{x}_{\boldsymbol{5}}$**=0.86,** $\boldsymbol{x}_{\boldsymbol{6}}$**=35.7.** | | **Observer 2** | | | | | |
|  |  | **+ s** | **+ c** | **N** | **- p** | **- r** | **Total** |
| **Observer 1** | **+ social** | 2 | 2 | 0 | 0 | 0 | 4 |
|  | **+ care** | 0 | 5 | 0 | 0 | 0 | 5 |
|  | **Neutral** | 0 | 1 | 0 | 0 | 0 | 1 |
|  | **- protective** | 0 | 0 | 0 | 2 | 0 | 2 |
|  | **- restrictive** | 0 | 0 | 0 | 0 | 0 | 0 |
|  | **Total** | 2 | 8 | 0 | 2 | 0 | 12 |
| **Observation period 15**  $\boldsymbol{x}_{\boldsymbol{1}}$**=C,** $\boldsymbol{x}_{\boldsymbol{2}}$**=4,** $\boldsymbol{x}_{\boldsymbol{3}}$**=14,** $\boldsymbol{x}_{\boldsymbol{4}}$**=9.68,** $\boldsymbol{x}_{\boldsymbol{5}}$**=2.05,** $\boldsymbol{x}_{\boldsymbol{6}}$**=37.5.** | | **Observer 2** | | | | | |
|  |  | **+ s** | **+ c** | **N** | **- p** | **- r** | **Total** |
| **Observer 1** | **+ social** | 2 | 5 | 0 | 0 | 0 | 7 |
|  | **+ care** | 1 | 17 | 1 | 1 | 0 | 20 |
|  | **Neutral** | 1 | 2 | 5 | 0 | 0 | 8 |
|  | **- protective** | 0 | 0 | 0 | 0 | 0 | 0 |
|  | **- restrictive** | 0 | 0 | 0 | 0 | 0 | 0 |
|  | **Total** | 4 | 24 | 6 | 1 | 0 | 35 |

**Supplementary table C^td^:**

| **Observation period 16**  $\boldsymbol{x}_{\boldsymbol{1}}$**=A,** $\boldsymbol{x}_{\boldsymbol{2}}$**=4,** $\boldsymbol{x}_{\boldsymbol{3}}$**=10,** $\boldsymbol{x}_{\boldsymbol{4}}$**=6.50,** $\boldsymbol{x}_{\boldsymbol{5}}$**=2.73,** $\boldsymbol{x}_{\boldsymbol{6}}$**=34.6.** | | **Observer 2** | | | | | |
| --- | --- | --- | --- | --- | --- | --- | --- |
|  |  | **+ s** | **+ c** | **N** | **- p** | **- r** | **Total** |
| **Observer 1** | **+ social** | 3 | 1 | 0 | 0 | 0 | 4 |
|  | **+ care** | 1 | 7 | 1 | 0 | 0 | 9 |
|  | **Neutral** | 0 | 0 | 3 | 0 | 0 | 3 |
|  | **- protective** | 0 | 1 | 0 | 1 | 0 | 2 |
|  | **- restrictive** | 0 | 0 | 0 | 0 | 0 | 0 |
|  | **Total** | 4 | 9 | 4 | 1 | 0 | 18 |
| **Observation period 17**  $\boldsymbol{x}_{\boldsymbol{1}}$**=B,** $\boldsymbol{x}_{\boldsymbol{2}}$**=4,** $\boldsymbol{x}_{\boldsymbol{3}}$**=9,** $\boldsymbol{x}_{\boldsymbol{4}}$**=9.09,** $\boldsymbol{x}_{\boldsymbol{5}}$**=1.93,** $\boldsymbol{x}_{\boldsymbol{6}}$**=30.** | | **Observer 2** | | | | | |
|  |  | **+ s** | **+ c** | **N** | **- p** | **- r** | **Total** |
| **Observer 1** | **+ social** | 4 | 3 | 0 | 0 | 0 | 7 |
|  | **+ care** | 1 | 7 | 0 | 0 | 0 | 8 |
|  | **Neutral** | 1 | 1 | 1 | 0 | 0 | 3 |
|  | **- protective** | 0 | 1 | 0 | 0 | 0 | 1 |
|  | **- restrictive** | 3 | 0 | 0 | 0 | 0 | 3 |
|  | **Total** | 9 | 12 | 1 | 0 | 0 | 22 |
| **Observation period 18**  $\boldsymbol{x}_{\boldsymbol{1}}$**=C,** $\boldsymbol{x}_{\boldsymbol{2}}$**=4,** $\boldsymbol{x}_{\boldsymbol{3}}$**=10,** $\boldsymbol{x}_{\boldsymbol{4}}$**=4.12,** $\boldsymbol{x}_{\boldsymbol{5}}$**=2.36,** $\boldsymbol{x}_{\boldsymbol{6}}$**=35.7.** | | **Observer 2** | | | | | |
|  |  | **+ s** | **+ c** | **N** | **- p** | **- r** | **Total** |
| **Observer 1** | **+ social** | 0 | 1 | 0 | 0 | 0 | 1 |
|  | **+ care** | 0 | 9 | 0 | 0 | 0 | 9 |
|  | **Neutral** | 0 | 0 | 2 | 0 | 0 | 2 |
|  | **- protective** | 0 | 0 | 0 | 0 | 0 | 0 |
|  | **- restrictive** | 0 | 0 | 0 | 0 | 0 | 0 |
|  | **Total** | 0 | 10 | 2 | 0 | 0 | 12 |

**1 KEY to observation period covariates:** $\boldsymbol{x}_{\boldsymbol{1}}$**=Ward,** $\boldsymbol{x}_{\boldsymbol{2}}$**=Number of occupied beds,**$\boldsymbol{x}_{\boldsymbol{3}}$**=Time of day (0 to 24 hours),** $\boldsymbol{x}_{\boldsymbol{4}}$**=Number of interaction per patient per hour,**$\boldsymbol{x}_{\boldsymbol{5}}$**=Mean time of interaction,**$\boldsymbol{x}_{\boldsymbol{6}}$**=Percentage of interactions of less than one minute.**
